# Comprehensive Serum Profiling for the Discovery of Epithelial Ovarian Cancer Biomarkers

**DOI:** 10.1371/journal.pone.0029533

**Published:** 2011-12-21

**Authors:** Ping Yip, Tzong-Hao Chen, Partha Seshaiah, Laurie L. Stephen, Karri L. Michael-Ballard, James P. Mapes, Brian C. Mansfield, Greg P. Bertenshaw

**Affiliations:** 1 Correlogic Systems, Inc., Germantown, Maryland, United States of America; 2 Rules-Based Medicine, Inc., Austin, Texas, United States of America; The University of Hong Kong, China

## Abstract

FDA-cleared ovarian cancer biomarkers are limited to CA-125 and HE4 for monitoring and recurrence and OVA1, a multivariate panel consisting of CA-125 and four additional biomarkers, for referring patients to a specialist. Due to relatively poor performance of these tests, more accurate and broadly applicable biomarkers are needed. We evaluated the dysregulation of 259 candidate cancer markers in serum samples from 499 patients. Sera were collected prospectively at 11 monitored sites under a single well-defined protocol. All stages of ovarian cancer and common benign gynecological conditions were represented. To ensure consistency and comparability of biomarker comparisons, all measurements were performed on a single platform, at a single site, using a panel of rigorously calibrated, qualified, high-throughput, multiplexed immunoassays and all analyses were conducted using the same software. Each marker was evaluated independently for its ability to differentiate ovarian cancer from benign conditions. A total of 175 markers were dysregulated in the cancer samples. HE4 (AUC = 0.933) and CA-125 (AUC = 0.907) were the most informative biomarkers, followed by IL-2 receptor α, α1-antitrypsin, C-reactive protein, YKL-40, cellular fibronectin, CA-72-4 and prostasin (AUC>0.800). To improve the discrimination between cancer and benign conditions, a simple multivariate combination of markers was explored using logistic regression. When combined into a single panel, the nine most informative individual biomarkers yielded an AUC value of 0.950, significantly higher than obtained when combining the markers in the OVA1 panel (AUC 0.912). Additionally, at a threshold sensitivity of 90%, the combination of the top 9 markers gave 88.9% specificity compared to 63.4% specificity for the OVA1 markers. Although a blinded validation study has not yet been performed, these results indicate that alternative biomarker combinations might lead to significant improvements in the detection of ovarian cancer.

## Introduction

Ovarian cancer is the most deadly gynecological cancer in the US with an estimated 21,880 new cases detected in 2010 [1]. When diagnosed and treated early, intervention is generally successful, with a 5-year survival rate of 93.5% [2]. Unfortunately, only 15% of ovarian cancers are found early, with the majority of cases detected at late stages where the outcome is far less favorable. For patients with distant malignancies, the 5-year survival rate is only 27.6%. As a result, approximately 14,000 women die each year from this cancer in the US [1]. Complicating diagnosis, ovarian cancer has an incidence of just 12.6 per 100,000 women [2]. Therefore, there is a pressing clinical need for a test that exhibits a high sensitivity for malignancies but also a high specificity to minimize the number of false positives that occur in such a low incidence disease.

Clinically, multiple lines of evidence are examined to assess the possibility of an individual having ovarian cancer. Typically, these include the presence of a pelvic mass, family history, and other symptoms (e.g. pelvic and abdominal pain, urinary urgency/frequency, abdominal bloating, and difficulty eating), supported by a physical examination, a radiographic evaluation, and laboratory findings. However, none of these assessments are specific for ovarian cancer and none differentiate well between cancerous and benign conditions [3]. Though radiographic evidence can help in the detection and diagnosis of a pelvic mass, the commonly used imaging techniques are interpreted subjectively and tend to have a low specificity in routine use [4]. Some reports suggest ultrasound alone or in combination with other prognostic variables may be significantly more informative in the hands of an ovarian ultrasound expert [5], [6]. However, many patients lack access to such specialized imaging services.

There are no US Food and Drug Administration (FDA)-cleared biomarkers for ovarian cancer screening. For the narrower application of monitoring disease recurrence and therapeutic response, two markers have been FDA-cleared: cancer antigen 125 (CA-125) in 1987, and more recently, human epididymis protein-4 (HE4) in 2008 [7], [8], [9], [10]. Despite this, CA-125 is frequently used off-label for initial diagnosis. However in this setting, the performance of CA-125 varies widely, depending on the cut-off selected, and the patient population, with sensitivities ranging from 29–100%. A further complication is that CA-125 gives many false positives in a wide variety of normal, benign and other malignancies, leading to low specificity [11], [12], [13].

Many approaches have been taken to improve the performance of CA-125. Improved specificity has been reported in a retrospective study using serial CA-125 measurements interpreted by a Risk of Ovarian Cancer Algorithm (ROCA). Initial reports suggest that the accuracy may be inadequate for initial diagnosis [14], although more definitive results are expected upon completion of a prospective clinical investigation in late 2011 [15]. Many other strategies have sought to combine CA-125 with additional markers [16], [17], [18], [19], [20]. OvaCheck® combines CA-125 with seven other markers and has 81.1% sensitivity and 85.4% specificity as determined in a double-blinded clinical validation study [21]. However, the test performance needs to be validated on a non-specialist population (e.g. obstetrician-gynecologist). The Risk of Ovarian Malignancy Algorithm (ROMA) combines measurements of both CA-125 and HE4 [22]. The Risk of Malignancy Index (RMI) attempts to improve specificity by combining CA-125 with an imaging score and menopausal status [23]. However, ROMA and RMI do not appear to increase performance significantly over CA-125 alone [24], [25], [26]. Another multimarker test, OvPlex™, which combines CA-125 with C-reactive protein, serum amyloid A (SAA), interleukin 6 (IL-6), and IL-8, was reported to have 94.1% sensitivity and 93.1% specificity [27]. However, case and control samples were not from the same patient population, raising significant concerns that selection biases inflated the reported performance. Similarly, a Yale developed test, OvaSure™, combines leptin, prolactin, osteopontin, insulin-like growth factor II and macrophage inhibitory factor with CA-125 and has a reported sensitivity of 95.3% and a specificity of 99.4% [28]. However multiple concerns about the study design and validation population have also challenged the validity of the markers selected and the significance of the reported performance [29], [30].

In 2009, an ovarian cancer multivariate test was FDA-cleared, but not for screening [31]. The test, based on the measurements of CA-125 with transthyretin, apolipoprotein AI, transferrin, and β2 microglobulin, was approved for the very limited population of women for whom surgery is already planned for an ovarian adnexal mass, when they have not yet been referred to an oncologist, and when the physician's independent clinical and radiological evaluation does not indicate malignancy. The performance of OVA1 depends on the source of the surgical patient population (specialist or non-specialist oncologist) and the menopausal status of the patient [31], [32]. For the intended use population (women in the care of a non-specialist and negative for malignancy by clinical assessment) the reported sensitivity was 70.0% (14/20) and the specificity 50.3% (82/163). For all patients under the care of a non-specialist, when OVA1 was added to clinical assessment, the reported sensitivity increased from 72.2% to 91.7% [31]. However, in conjunction with the 19.5% increase in sensitivity, there was a dramatic 41.1% decrease in specificity (82.7% to 41.6%) and an associated 24.0% decrease in PPV (60.5% to 36.5%).

In a recent series of publications involving multiple groups coordinated by the Early Detection Research Network (EDRN) of the US National Cancer Institute (NCI), it was shown that for early detection, 49 promising biomarkers could not improve the performance over CA-125, whether alone or in combinations [33], [34], [35]. In conclusion, simple, broadly applicable, clinical tests for the detection of ovarian cancer remain elusive, and there is a need for a wider search for novel, informative, cancer markers and combinations. In a previous report, we profiled 104 common serum biomarkers, 44 autoimmune markers and 56 infectious-disease markers [36] and reported on their individual ability to discriminate ovarian cancer from normal and benign conditions. In related studies, we built panels of biomarkers to improve the performance of the individual biomarkers ([21], [37]). The final panel, OvaCheck, was intended to be used to refer women with symptoms of ovarian cancer to a gynecologic oncologist. We hypothesized that by profiling additional biomarkers, we might discover new and informative biomarkers, that could be used to modify the existing OvaCheck panel and improve its overall performance. We proposed to address this in two distinct steps. First, in the current study, we have extended the biomarker discovery work, with an additional 155 biomarkers discovered primarily through cancer research. In the second step, yet to be undertaken, we will evaluate the modified panel, using a new, prospectively collected, blinded validation set of samples. We now report on a 259 serum biomarker survey of almost 500 new patients with ovarian cancer or benign conditions. Our samples were drawn from a large, prospective clinical study of adnexal masses performed across 11 independent sites, with sera drawn under a uniform protocol, prior to surgical intervention, and prior to knowledge of disease status. To ensure consistency, all assays and samples were performed on a single platform at a single site and all analyses were conducted using the same software. We have analyzed the data for evidence of useful single markers of ovarian cancer across disease subtype and stage. Our findings point to the absence of a single diagnostic marker, and support the increasing emphasis on the development of multivariate assays using logistic regression or more sophisticated algorithms.

## Materials and Methods

### Study Cohort

Sera were from a prospective collection undertaken by Correlogic Systems, Inc. specifically to develop and validate the performance of an ovarian cancer test [21]. All samples were collected under a uniform protocol from 11 different sites, which were monitored for adherence. The Western Institutional Review Board (Olympia, WA) and the IRBs of the individual sites approved the studies under FDA Investigational Device Exemption (IDE) number G050132. The collection sites (and IRBs) were: Cedars-Sinai Medical Center, Los Angeles, CA (Cedars-Sinai Institutional Review Board); Florida Gynecologic Oncology, Fort Meyers, FL (Lee Memorial Health System Institutional Review Committee); Florida Hospital Cancer Institute, Orlando, FL (Florida Hospital Institutional Review Board); The Harry and Jeanette Weinberg Cancer Institute at Franklin Square Hospital, Baltimore, MD (MedStar Research Institute Georgetown Oncology Institutional Review Board); Holy Cross Hospital, Silver Spring, MD (Holy Cross Institutional Review Board); North Shore – Long Island Jewish Health System, Manhasset, NY (Institutional Review Board North Shore-Long Island Jewish Health System); SUNY at Stony Brook, NY, Stony Brook, NY (Committee on Research Involving Human Subjects SUNY Stony Brook); University of Alabama at Birmingham, Birmingham, AL (The University of Alabama at Birmingham Institutional Review Board for Human Use); University of Southern California, Norris Cancer Center, Los Angeles, CA and Women's and Children's Hospital, Los Angeles, CA (University of Southern California Health Sciences Campus Institutional Review Board); Wake Forest University Health Sciences, Winston-Salem, NC (Institutional Review Board Wake Forest University School of Medicine); and Women and Infants Hospital of Rhode Island, Providence, RI (Institutional Review Board Women and Infants' Hospital of Rhode Island). The study inclusion criteria were women, at least 18 years of age, symptomatic of ovarian cancer according to the National Comprehensive Cancer Network (NCCN) Ovarian Cancer Treatment Guidelines for Patients [3], which includes women with or without a pelvic mass. Participants had to be scheduled for gynecologic surgery based on concern they had ovarian cancer, and post-surgical pathological evaluation of the ovaries and excised tissues was required to establish clinical truth of disease status. Exclusion criteria were women who did not meet the inclusion criteria, could not provide informed consent, were pregnant, or previously treated for ovarian cancer. Written informed consent was obtained for each participant in the study. All data were de-identified and no results were returned to the physicians or patients.

In the present study, we utilized 149 samples from the patients with pathology-confirmed ovarian cancer and 350 samples from the patients with pathology-confirmed benign conditions (Table 1). The ovarian cancer samples included all stages and common subtypes of the disease. The benign samples included the common types of benign conditions seen in the entire study population. Complete clinicopathology reports, obtained following surgery, along with the patient age, race, staging, subtype and coded collection site accompanied each sample.

**Table 1:** Demographics of the Study Subjects.

	Ovarian cancer - FIGO Stage and Subtype						Benign
	I	II	III	IV	X	All	--
**Number of Samples (%):**							
**Ovarian Cancer**							
**Serous**	8	10	60	4	0	82 (55.0)	--
**Mucinous**	7	0	1	1	0	9 (6.0)	--
**Clear Cell**	9	1	3	1	0	14 (9.4)	--
**Endometrioid**	5	4	4	0	0	13 (8.7)	--
**Brenner**	1	0	0	1	0	2 (1.3)	--
**Poorly/undifferentiated**	0	0	2	1	0	3 (2.0)	--
**Mixed**	10	2	13	0	1	26 (17.4)	--
**Total**	40	17	83	8	1	149	--
	(26.8)	(11.4)	(55.7)	(5.4)	(0.7)	(100)	--
**Benign**							
**Neoplastic**	--	--	--	--	--	--	105 (30.0)
**Non-neoplastic**	--	--	--	--	--	--	155 (44.3)
**Mixed**	--	--	--	--	--	--	64 (18.3)
**No abnormalities**	--	--	--	--	--	--	26 (7.4)
**Total**							350 (100)
**Population Age:**							
**Median age (years)**	59	61	63	68	76	61	51
**Range (years)**	47-88	33-85	30-84	55-80	76-76	30-88	18-85
**Mean age (years)**	61.4	60.5	62.7	67.1	76.0	62.4	52.8
**SD**	10.5	13.7	11.5	9.6	--	11.4	14.0

The neoplastic conditions seen were: Adenofibroma; Brenner tumor (benign); Cyst-thecoma; Cystadenofibroma; Cystadenofibroma-mucinous; Cystadenofibroma-serous; Cystadenoma; Cystadenoma-mucinous; Dermoid cyst; Fibroadenoma-serous; Fibrothecoma; Firbroma; Struma ovarii; Teratoma-cystic/mature/immature. The non-neoplastic conditions seen were: Adhesions; Atrophic ovary/atrophic changes; Calcifications; Calcified corpus albicans; Cyst-epithelial/benign/simple; Cyst-epithelial inclusion/celomic inclusion/mullerian inclusion/inclusion; Cyst-hemorrhagic follicular/corpus luteal/follicular/corpus albicans; Cyst-serous; Cyst-theca-lutein; Endometrioma/endometriosis/endometriotic cyst; Endosalpingiosis; Endosalpingiosis; Fibrosis; Focal surface mesothelial hyperplasia; Hemorrhagic corpus luteum/corpus luteum/corpora abicantia; Hemorrhagic ovary; Hyperthecosis - stromal; Psammoma bodies; Surface micropapillary/stromal proliferation; Torsion; Tubo-ovarian abscess; Tubo-ovarian adhesion. Abbreviations: FIGO, International Federation of Gynecology and Obstetrics; SD, standard deviation; X, staging not available.

### Serum Processing, Storage, Handling and Shipment

Prior to any intervention, blood samples (10 ml) were collected into red top glass Vacutainer tubes. The blood was clotted for at least 30 minutes at room temperature, centrifuged at 3,500 g for 10 minutes, and the resulting serum removed into pre-labeled cryotubes, and stored promptly at −80°C. Processing from blood draw to freezing was completed within 2 hours. All samples were shipped on dry ice to a single designated site for storage. To aliquot, all samples were thawed in a water and ice slurry then transferred into sample tubes labeled with coded identifiers that blinded all subsequent experimenters to the sample disease status. Samples were then shipped on dry ice to Rules-Based Medicine, Inc. (RBM; Austin, TX) for assaying. An accompanying document provided the list of coded sample identifiers and specified an order of analysis designed to remove any sample type position bias during analysis. As a result of these precautions, the RBM analytical site was completely blinded to cancer status, pathology and all other sample details.

### Multiplex Immunoassays

Two hundred and fifty nine serum biomarkers were measured using a set of proprietary multiplexed immunoassays (Human DiscoveryMAP® v1.0 and Human OncologyMAP® v1.0; Table S1) at RBM in their Luminex-based CLIA-certified laboratory. Each assay was calibrated using an 8-point standard curve, performed in duplicate. Median Fluorescence Intensity (MFI) measurements were interpolated into final protein concentrations using RBM's proprietary curve-fitting software. Assay performance was verified using quality control (QC) samples at low, medium and high levels for each analyte in duplicate. All standard and QC samples were in a complex serum-based matrix to match the sample background matrix. Since sera were analyzed at a previously optimized dilution, any reading above the maximum concentration of the calibration curve was assigned the concentration of the highest standard, whereas any below the minimum concentration was assigned the value 0. For analysis, the sample run order was randomized to avoid any sequential bias due to presence or absence of disease, subtype or stage of disease, patient age, or age of serum sample.

### Data Analysis

Descriptive statistics, Receiver Operating Characteristic (ROC) curves and graphical displays (dot plots) for serum analyte concentrations were performed using GraphPad Prism version 5.0a (GraphPad Software, Inc., San Diego, CA). Statistical differences were determined using the nonparametric Kruskal-Wallis test (ANOVA) followed by Dunn's multiple comparison post-test. For all statistical comparisons a P-value *<*0.05 was interpreted as statistically significant. A Pearson correlation matrix was created using the proprietary multi-spectral analysis application SpectraViewer™ (Correlogic Systems).

### Logistic Regression

Multivariate models were built by logistic regression using an in-house developed python script.

## Results and Discussion

Using multiplexed immunoassays, we measured simultaneously the levels of 259 molecules in sera from 149 patients with pathology-confirmed epithelial ovarian cancer and 350 individuals with benign ovarian conditions (Table 1). Since we were interested in the ability of biomarkers to differentiate between symptomatically similar cancer and benign gynecological conditions, all samples were obtained from the same clinical population – women presenting for surgery primarily based on the presence of an adnexal mass. All samples were collected before any intervention and before the disease status was known. Disease status was subsequently identified by pathology exams of the excised tissue. Sera were collected using a single sample collection protocol that was monitored for compliance. The study was conducted prospectively at 11 sites that were also monitored for protocol adherence. This assured sample quality and removed the possibility of any collection, processing or biological biases in the sample set, a concern for many other studies [30]. No normal healthy samples were used in this study, as they are typically easier to classify than benign conditions [21] and introduce confounding factors such as lower stress levels compared to patients facing surgery [38]. As expected, the median patient age was higher in individuals with ovarian cancer (61 years) than those with benign conditions (51 years) and increased with the stage of disease present (Table 1; [28], [36]). The distribution of the ovarian cancer subtypes was similar to the distribution seen for all ovarian cancer cases in the US population as a whole, with a larger proportion of serous carcinoma (55%) than other subtypes (Table 1). The benign controls in the study were representative of common benign ovarian conditions including cystadenoma, cystadenofibroma and fibroma.

To ensure consistency and aid in biomarker comparisons, all 259 markers and 499 samples were measured on a single platform at a single site using a panel of rigorously qualified, high-throughput, multiplexed immunoassays. This survey built on our previous profiling of 104 serum biomarkers [36]. The majority of the additional 155 serum biomarkers in the present study were developed as part of two NCI-funded Small Business Innovative Research (SBIR) awards specifically targeted at markers that had reasonable literature support to suggest a significant role in cancer biomarker. The selected biomarkers covered a broad range of biological functions, primarily implicated in cancer including cancer antigens, hormones, clotting factors, tissue modeling factors, lipoprotein constituents, proteases and protease inhibitors, markers of cardiovascular risk, growth factors, cytokine/chemokines, soluble forms of cell-signaling receptors, and inflammatory and acute phase reactants (Table S1). To our knowledge, the present study is the broadest and most consistent single study of immunoassay profiling of molecules using fully characterized, quality-controlled samples.

**Figure 1:**
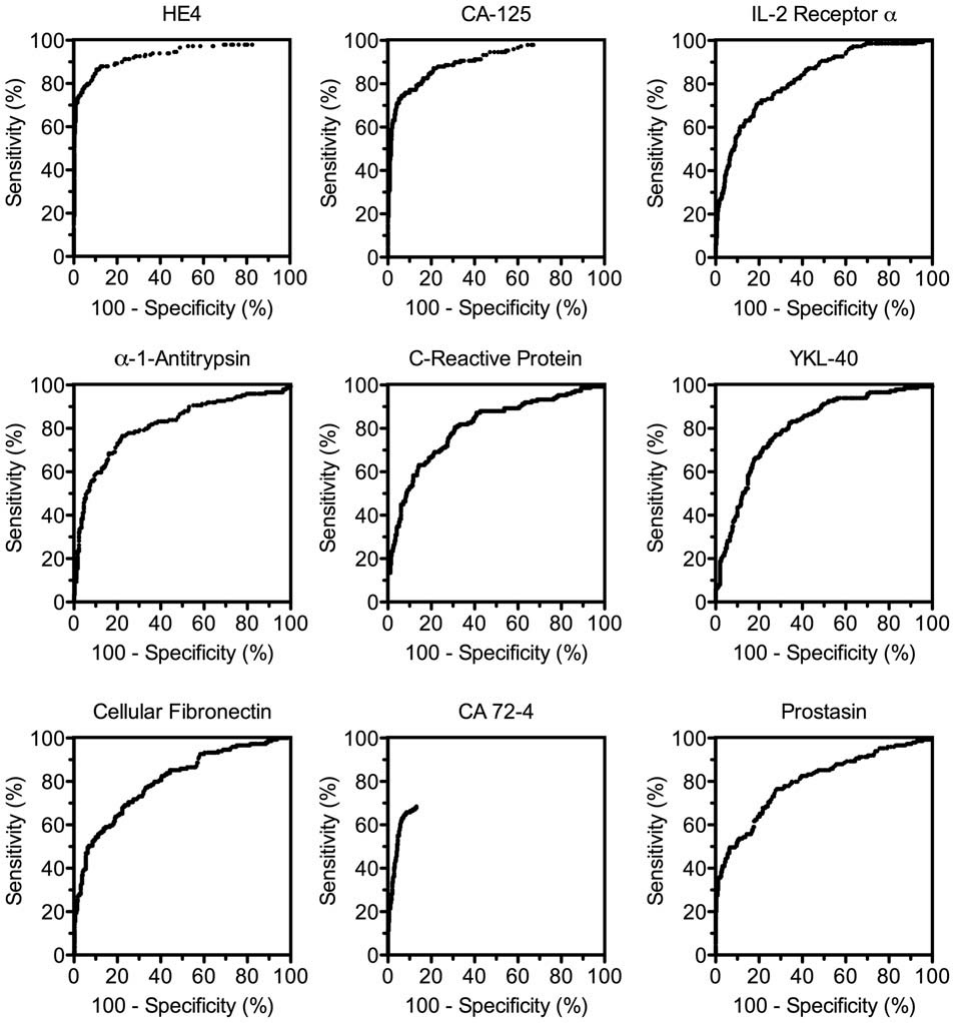
Receiver Operating Characteristic curves for the nine most informative biomarkers with area underneath the curve values greater than 0.800.

For each biomarker, an ROC curve was generated and its area under the curve (AUC) value compared to that of an uninformative marker (AUC = 0.500). A total of 175 biomarkers were dysregulated (P-values>0.05) in the ovarian cancer samples relative to the benign gynecological conditions. Of these, 136 biomarkers were up-regulated and 39 down-regulated (Table 2 and Table S2). The biomarkers with the greatest AUC values were predominantly up-regulated in ovarian cancer (Table 2, Fig. 1 and Table S2) with values ranging from 0.599 to 0.933. The most up-regulated markers were, not surprisingly, HE4 and CA-125 with AUC values of 0.933 and 0.907, respectively, followed by interleukin-2 receptor α (IL-2 receptor α), α1-antitrypsin, C-reactive protein, YKL-40, cellular fibronectin, cancer antigen 72-4 (CA-72-4) and prostasin, with AUC values between 0.829 and 0.800 (Table 2). The remaining 127 up-regulated biomarkers had a continuum of AUC values from 0.797 to 0.556 (Table S2). Thirty-four of the remaining 127 markers had AUC values above 0.700. For down-regulated biomarkers, the AUC values ranged from 0.556 to 0.745 (Table S2). The two most informative of these stood out as transthyretin (0.745) and apolipoprotein A-IV (0.713), while the remaining biomarkers had AUC values below 0.700.

**Table 2:** Area Underneath the Curve (AUC) values from Receiver Operating Characteristic (ROC) curve analysis of the top 20 markers.

Marker	AUC	Std. Error	95% CI
**HE4**	0.933	0.014	0.905 to 0.961
**CA-125**	0.907	0.016	0.877 to 0.938
**IL-2 receptor alpha**	0.829	0.020	0.790 to 0.868
**Alpha-1-antitrypsin**	0.817	0.023	0.773 to 0.861
**C-reactive protein**	0.806	0.022	0.763 to 0.850
**YKL-40**	0.804	0.021	0.763 to 0.845
**Cellular Fibronectin**	0.803	0.022	0.760 to 0.846
**CA-72-4**	0.802	0.025	0.753 to 0.850
**Prostasin**	0.800	0.023	0.755 to 0.845
**TIMP-1**	0.797	0.024	0.751 to 0.844
**IL-8**	0.795	0.022	0.752 to 0.837
**MMP-7**	0.787	0.024	0.741 to 0.834
**IL-6**	0.786	0.024	0.740 to 0.833
**VEGF-B**	0.767	0.024	0.720 to 0.815
**Calprotectin**	0.767	0.024	0.719 to 0.814
**IGFBP-2**	0.759	0.023	0.714 to 0.805
**LOX-1**	0.750	0.023	0.704 to 0.796
**Neuropilin-1**	0.750	0.024	0.702 to 0.798
**TNFR2**	0.748	0.024	0.700 to 0.796
**MPIF-1**	0.745	0.025	0.697 to 0.793

NOTE: The P value was <0.0001 for all markers and tested the null hypothesis that Area Underneath the Curve (AUC) = 0.5. Abbreviations: 95% CI, 95% confidence interval; HE4, human epididymis protein-4; CA, cancer antigen; TIMP-1, tissue inhibitor of metalloproteinases 1; IL, interleukin; MMP-7, Matrix Metalloproteinase-7; VEGF-B, vascular endothelial growth factor B; IGFBP-2, insulin-like growth factor-binding protein 2; LOX-1, lectin-like oxidized LDL receptor 1; TNFR2, tumor necrosis factor receptor 2; MPIF-1, myeloid progenitor inhibitory factor 1.

Thirteen of the twenty biomarkers with the highest AUC values, namely HE4, IL-2 receptor α, YKL-40, cellular fibronectin, CA 72-4, prostasin, MMP-7, VEGF-B, Calprotectin, IGFBP-2, LOX-1, neuropilin-1 and MPIF-1 were not present in our previous study (Table 2; [36]). Therefore, the literature-based selection criteria for biomarkers appears to have been successful. While implicated in cancers before [22], [39], this is the first time that these molecules have been accurately quantified together, on a coherent set of samples, under uniformly controlled analytical conditions, to determine their discriminative power for ovarian cancer. We believe this approach improves biomarker comparisons and should aid in the selection of biomarkers in the development of multi-biomarker panels.

We compared the informativeness of the biomarkers that were measured in both our current and previous study. CA-125 was the most informative biomarker that was measured in both studies, and had remarkably similar AUC values of 0.907 (current) and 0.906 (previous). However, while the same biomarkers emerged in both studies as informative, AUC values across the studies varied. For example, the most informative biomarkers, α-1-antitrypsin (0.817 versus 0.642), C-reactive protein (0.806 versus 0.756), TIMP-1 (0.797 versus 0.701), IL-8 (0.795 versus 0.717), IL-6 (0.786 versus 0.693) and TNFR2 (0.748 versus 0.625) all had AUC values that were considerably higher in the current study. In addition, IL-10 (0.665 versus 0.725), EGFR (0.635 versus 0.733) and insulin (0.626 versus 0.671) had AUC values that were considerably lower in the current study. There are three potential reasons for the differences in AUC values. First, the two studies used different patient samples from different sources. The previous study used samples from the National Cancer Institute–funded Gynecologic Oncology Group (GOG) whereas in the current study we used samples from our own prospective collection, which may also have been more actively monitored for strict protocol compliance. Second, the non-ovarian cancer samples in the previous study were not completely restricted to benign conditions but also included samples from normal healthy individuals (19.7%) and individuals with other cancers (9.5%). Third, the sample preparation methods differed, the GOG samples were clotted on ice as opposed to room temperature in the current study, a difference known to be important for consistent levels of serum markers [40], [41]. The differences seen between the studies highlight the critical importance of complete control of sample collection, handling and population selection when performing and interpreting biomarker studies.

As a comparison between the two most informative biomarkers in this study, the sensitivity for HE4 and CA-125 was determined over a range of specificity values. In addition, the optimal cut-off value, defined as that yielding the greatest sum of specificity and sensitivity was calculated for each biomarker. The sensitivity for HE4 alone decreased from 89.0% to 57.1% as specificity increased from 80% to 99.6%, while for CA-125 alone the sensitivity decreased from 85.2% to 30.2%. The optimal cut-off for HE4 and CA-125 was 54.8 pM and 52.5 U/mL, respectively giving sensitivity values of 86.6% and 74.5%, respectively, and specificity values of 89.4% and 93.7%, respectively. As expected from ROC curves, there are trade-offs when no individual biomarker shows high specificity at a predetermined high sensitivity value. For example, at 100% sensitivity, both HE4 and CA-125 were 0% specific. At 98% sensitivity, HE4 had 30.6% specificity and CA-125 had 35.4% specificity. However, to see relatively good specificity values, the sensitivities had to be lowered to approximately 95%. At 95% sensitivity, HE4 had 50.9% specificity and CA-125 had 45.4% specificity. These values, along with the AUC values, indicated that on this population, HE4 performed slightly better than CA-125. In addition, these results show that none of the biomarkers in this study are sufficiently informative as standalone ovarian cancer biomarkers for broad applications and that biomarker panels may be needed to improve performance to clinically acceptable levels.

**Figure 2:**
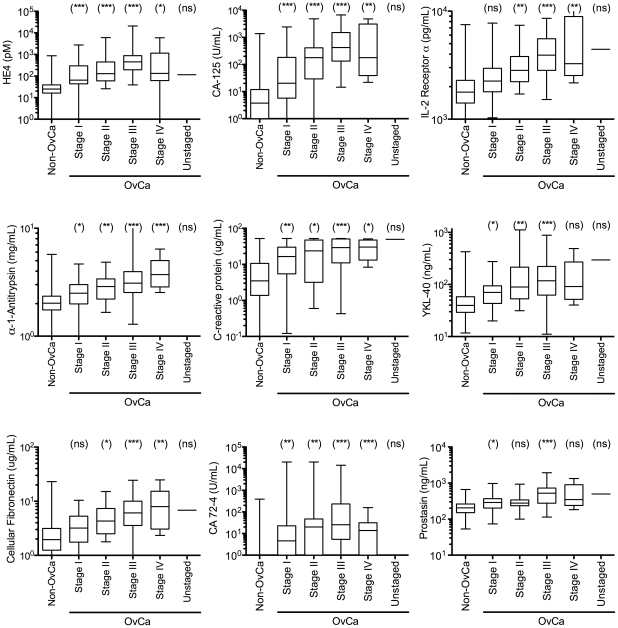
Serum level distributions broken out by International Federation Of Gynecology And Obstetrics (FIGO) ovarian cancer stage for the nine most informative biomarkers with area underneath the curve values greater than 0.800. The box boundaries represent the 25th and 75th percentiles, and the bar within the box represents the median value. The minimum and maximum values are represented by the extremes of the whiskers. ***, P-value<0.001; **, P-value 0.001-0.01; *, P-value 0.01–0.05; ns, P-value>0.05. Abbreviations: OvCa, ovarian cancer; Non-OvCa, non-ovarian cancer; CA, cancer antigen; IL, interleukin.

To determine if some biomarkers might have greater discrimination for different stages of cancer, especially early stage, we compared the nine biomarkers with AUC values above 0.800 on FIGO stage I and II samples where there is the greatest need for marker-based detection (Fig. 2). For FIGO stage I samples, both HE4 and CA-125 were highly discriminative (P-values<0.001), followed in descending order by C-reactive protein and CA 72-4 (P-values 0.001–0.01) then α1-antitrypsin, YKL-40 and prostasin (P-values 0.01–0.05). For IL2-receptor α and cellular fibronectin, there were no statistical differences between stage I cancer and benign conditions (P-values>0.05). For FIGO stage II samples, both HE4 and CA-125 were again highly discriminative (P-values<0.001), followed by for IL2-receptor α, α1-antitrypsin, YKL-40 and CA 72-4 (P-values 0.001–0.01) and then C-reactive protein and cellular fibronectin (P-values 0.01–0.05). For prostasin, there was no statistical difference (P-value>0.05).

**Figure 3:**
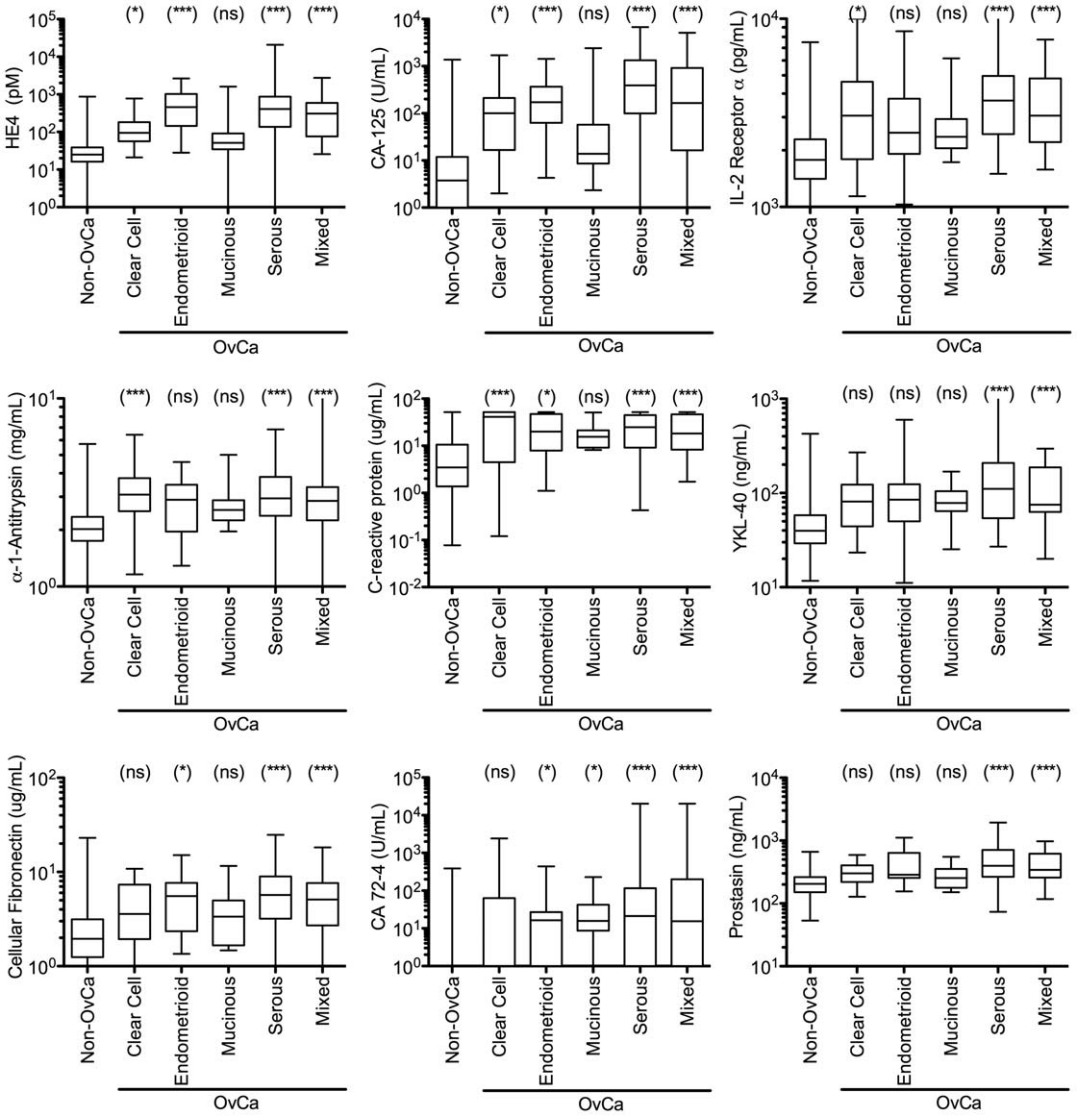
Serum level distributions broken out by subtype of ovarian cancer stage for the nine most informative biomarkers with area underneath the curve values greater than 0.800. The box boundaries represent the 25th and 75th percentiles, and the bar within the box represents the median value. The minimum and maximum values are represented by the extremes of the whiskers. ***, P-value<0.001; **, P-value 0.001–0.01; *, P-value 0.01-0.05; ns, P-value>0.05. Abbreviations: OvCa, ovarian cancer; Non-OvCa, non-ovarian cancer; CA, cancer antigen; IL, interleukin.

For the same nine biomarkers we also determined if there were statistically significant differences between samples from women with benign conditions and women with each individual subtype of ovarian cancer (Fig. 3). For clear cell carcinomas, α1-antitrypsin and C-reactive protein were highly discriminatory (P-values<0.001), followed in descending order by HE4, CA-125 and IL2-receptor α (P-values 0.01–0.05). For YKL-40, cellular fibronectin, CA 72-4 and prostasin there were no statistical differences (P-value>0.05). For endometrioid carcinomas, there were highly significant differences for HE4 and CA-125 (P-values<0.001) and significant differences for C-reactive protein, cellular fibronectin, CA 72-4 (P-values 0.01–0.05). For α1-antitrypsin, IL2-receptor α, YKL-40 and prostasin there were no statistical differences (P-values>0.05). For mucinous carcinomas, only CA 72-4 had a significant difference (P-value 0.01–0.05). For serous and mixed carcinomas, all nine biomarkers had highly significant differences (P-value<0.001). Therefore, with the exception of mucinous carcinomas, the nine biomarkers are informative for all common ovarian cancer subtypes, however, their different discriminative powers suggests that different combinations of markers may be useful for different subtypes. While it would have been preferential to find more informative biomarkers for the mucinous subtype, it is relatively rare. Indeed, only 6.0% of the cancers in the study were of mucinous subtype (Table 1).

**Figure 4:**
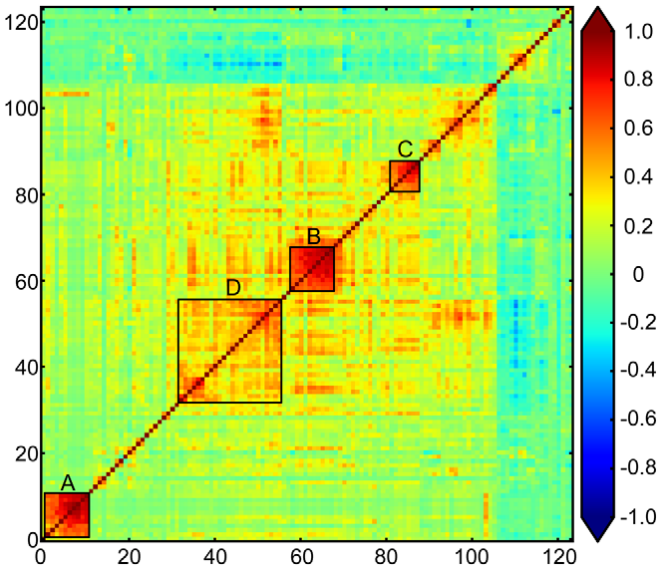
Correlation matrix for biomarkers with area underneath the curve values greater than 0.600. Hierarchical clustering was implemented with Pearson's correlation coefficients as the distance measure. Intense red and blue colors, signify strong positive and negative correlation, respectively.

For simplicity and cost effectiveness, the use of a single biomarker is preferred over multiple biomarkers. However, it is clear that single biomarkers may not be able to capture the inherent diversity of complexes diseases such as ovarian cancer. An informative test seeks to combine multiple biomarkers in a way that each marker adds a different type of discrimination either to the entire patient population or the population subdivisions made by the other markers. Simply put, markers with poor correlation with one another have a greater chance of individually contributing to a panel than markers with strong correlation with one another. Therefore, we performed correlation analysis on the strongest ovarian cancer markers - the 124 biomarkers with AUC values greater than 0.600. The co-varying molecules were sorted agglomeratively with hierarchical clustering using Pearson correlation coefficients as the distance measure. The pair-wise results were assembled into a 124×124 matrix (numbered 0–123) and displayed using a heat map where an intense red color signifies strong positive correlation and blue signifies a negative correlation (Fig. 4). There were four major clusters (Clusters A through D; Fig. 4; Tables S3, S4, S5, S6, and S7), each cluster representing markers strongly correlating with each other. Each of these clusters contained markers that are strong ovarian cancer markers. Cluster A (markers 1–10) contained two strong ovarian cancer markers, CA 72-4 and MPIF-1 (Tables S3 and S4). TNFR2 was found in Cluster B (markers 58–67; Tables S3 and S5). Cluster C (markers 79–87) contained the two strongest ovarian cancer markers (HE4, CA-125) as well as prostasin and VEGF-B (Tables S3 and S6). The strongest correlations with CA-125 were mesothelin (Pearson correlation coefficient = 0.600), maspin (0.599), VEGF-D (0.568), prostasin (0.551), kallikrein-7 (0.507) and VEGF-B (0.505). Maspin (0.517) correlated with HE4 the strongest, followed by TIMP-1 (0.470), prostasin (0.463), IL-2 receptor α (0.424), VEGF-B (0.413) and VEGF-D (0.409). Finally, the largest cluster (Cluster D; biomakers 32–55), was composed of loosely correlated markers that contained several good ovarian cancer markers including calprotectin, LOX-1, IL-6, YKL-40, cellular fibronectin, neuropilin-1, α1-antitrypsin, TIMP-1, C-reactive protein and IL-2 receptor α (Tables S3 and S7). These correlation data can help drive the development of biomarkers panels and may give insights into pathways that are disrupted in ovarian cancer.

To examine how the information in this study can be used, we studied a simple multi-marker scenario where we measured the combined performance of the nine markers with AUC values greater than 0.800. The nine markers were combined using logistic regression which yielded an AUC of 0.950 (Standard error: 0.01213; 95% CI: 0.926–0.974; P-value: <0.0001). Next we compared this performance against the five markers in the FDA-cleared OVA1 test. The samples in our study were collected at gynecologic oncologists. A similar study population was reported in the OVA1 510(k) summary with 100% sensitivity (invasive ovarian cancer only) and 32.9% specificity [31]. We combined the five markers and built a logistic regression model. Consistent with the OVA1 510(k) summary, with our sample set, at 32.9% specificity, OVA1 biomarkers gave a sensitivity of 98.0%. Interestingly, with our samples, at a specificity of 32.9%, CA-125 alone had a sensitivity of 98.0%. This indicated that the additional OVA1 markers contributed little, if any, to the overall classification. Indeed, the AUC value for the five OVA1 biomarkers was 0.912 (Standard error: 0.0157; 95% CI: 0.881–0.943; P-value: <0.0001), barely higher than CA-125 alone which had an AUC of 0.907 (Standard error: 0.01571; 95% CI: 0.877–0.938; P-value: <0.0001). We compared the two models further by determining the sensitivity of models at fixed specificity values and the specificity of models at fixed sensitivity values (Tables 3 and 4). In general, the logistic regression model built on the top 9 markers outperformed the model built on OVA1 markers at all points of the ROC curve. At fixed specificity values between 80 and 95%, the top 9 model was 8 to 10% more sensitive that the model built on the OVA1 markers. At higher specificity (99%), the top 9 model was approximately 19% more sensitive. At fixed sensitivity between 80 and 99%, the top 9 model was between 8 and 25% more specific than the model built on the OVA1 markers.

**Table 3:** Sensitivity at Landmark Threshold Specificity Values of Logistic Regression Models using the Nine Most Informative and OVA1 Biomarkers.

	Sensitivity (%)	
Threshold Specificity (%)	Top 9	OVA1
**80**	92.6	84.6
**90**	88.6	79.2
**95**	83.9	73.2
**99**	69.1	50.3

Top 9, a logistic regression model was built using the 9 markers which had the highest individual AUC values; OVA1, a logistic regression model was built using the 5 markers in the OVA1 panel.

**Table 4:** Specificity at Landmark Threshold Sensitivity Values of Logistic Regression Models using the Nine Most Informative and OVA1 Biomarkers.

	Specificity (%)	
Threshold Sensitivity (%)	Top 9	OVA1
**80**	96.3	88.3
**90**	88.9	63.4
**95**	62.3	52.9
**99**	20.6	7.1

Top 9, a logistic regression model was built using the 9 markers which had the highest individual AUC values; OVA1, a logistic regression model was built using the 5 markers in the OVA1 panel.

In addition to the simple logistic regression approach described above, it will be interesting to utilize the correlation data from this study to help build more accurate multivariate models. For example, a panel composed of the strongest markers from each cluster as well as markers that were not found in any of the clusters, such as IL-8, MMP-7 and IGFBP2 may be even more accurate. However, these types of analyses are extensive, with many possible combinations to characterize, are beyond the scope of this initial publication and will form the basis of future work.

As both the top nine and OVA1 panels contained markers that may perform differently for pre- and post-menopausal women, we separately analyzed the performance of the two panels by menopausal status. For the top nine panel, the AUC value for pre-menopausal women was lower (0.937) than for post-menopausal women (0.953). This is consistent with the individual marker analysis that demonstrated that the top three individual markers (HE4, CA-125 and IL2-Rα) all performed better for the post-menopausal women (0.927, 0.927 and 0.824, respectively; Table S8) than for the pre-menopausal women (0.912, 0.907 and 0.812, respectively). For the OVA1 panel, the AUC value for pre-menopausal women was slightly lower (0.920) than for post-menopausal women (0.924). Again, this is consistent with the individual marker analysis that demonstrated that CA-125, the marker that appears to drive the performance of the OVA1 panel, performed worse for the group of pre-menopausal women (0.907) than for post-menopausal women (0.927).

In conclusion, we have identified new biomarkers that are capable of discriminating between samples drawn from women with benign ovarian conditions and those from women with ovarian cancer. Preliminary multivariate analysis, using a logistic regression model on the nine most informative biomarkers appeared to have significantly improved performance over the OVA1 biomarkers. Our analysis indicates that our data have the potential to improve on OVA1 and other tests. However, our study does not include a blinded validation set of samples. Therefore, we plan additional assessments on other, independent, well-characterized sample sets to independently validate these findings.

## Supporting Information

Table S1 **Biomarkers Assayed in Study.**
(DOC)

Table S2 **Informative Biomarkers with Area Underneath the Curve (AUC) Values Statistically Greater than 0.5.**
(DOC)

Table S3 **Identity of Markers in Clusters A Through D.**
(DOC)

Table S4 **Correlation of Markers in Cluster A.**
(DOC)

Table S5 **Correlation of Markers in Cluster B.**
(DOC)

Table S6 **Correlation of Markers in Cluster C.**
(DOC)

Table S7 **Correlation of Markers in Cluster D.**
(DOC)

Table S8 **Area Underneath the Curve (AUC) values from Receiver Operating Characteristic (ROC) curve analysis of the top 20 markers broken out by menopausal status.**
(DOC)
