# Supplementary material for: Comprehensive Serum Profiling for the Discovery of Epithelial Ovarian Cancer Biomarkers
Source: PLoS One. 2011 Dec 21;6(12):e29533. doi: 10.1371/journal.pone.0029533 (PMC3244467; doi:10.1371/journal.pone.0029533)
Supplement: Table S1 — Biomarkers Assayed in Study. (DOC) [file pone.0029533.s001.doc]

**Supplementary Table 1: Biomarkers Assayed in Study.**

| 6Ckine; Adiponectin; Agouti-Related Protein (AGRP); Aldose Reductase; Alpha-1-Antichymotrypsin (AACT); Alpha-1-Antitrypsin (AAT); Alpha-1-Microglobulin (A1Micro); Alpha-2-Macroglobulin (A2Macro); Alpha-Fetoprotein (AFP); Amphiregulin (AR); Angiogenin; Angiopoietin-2 (ANG-2); Angiotensin-Converting Enzyme (ACE); Annexin A1 (ANXA1); Apolipoprotein A-I (Apo A-I); Apolipoprotein A-II (Apo A-II); Apolipoprotein A-IV (Apo A-IV); Apolipoprotein B (Apo B); Apolipoprotein C-I (Apo C-I); Apolipoprotein C-III (Apo C-III); Apolipoprotein D (Apo D); Apolipoprotein E (Apo E); Apolipoprotein H (Apo H); Apolipoprotein(a) (Lp(a)); AXL Receptor Tyrosine Kinase (AXL); B cell-activating factor (BAFF); B Lymphocyte Chemoattractant (BLC); Bcl-2-like protein 2 (Bcl2-L-2); Beta-2-Microglobulin (B2M); Betacellulin (BTC); Bone Morphogenetic Protein 6 (BMP-6); Brain-Derived Neurotrophic Factor (BDNF); Calbindin; Calcitonin; Calprotectin; Cancer Antigen 125 (CA-125); Cancer Antigen 15-3 (CA-15-3); Cancer Antigen 19-9 (CA-19-9); Cancer Antigen 72-4(CA-72-4); Carcinoembryonic Antigen (CEA); Cathepsin D; CD 40 antigen (CD40); CD40 Ligand (CD40-L); CD5 (CD5L); Cellular Fibronectin (cFib); Chemokine CC-4 (HCC-4); Chromogranin-A (CgA); Ciliary Neurotrophic Factor (CNTF); Clusterin (CLU); Collagen IV; Complement C3 (C3); Complement Factor H; Connective Tissue Growth Factor (CTGF); Cortisol (Cortisol); C-peptide; C-Reactive Protein (CRP); Creatine Kinase-MB (CK-MB); Cystatin-C; Endoglin; Endostatin; Endothelin-1 (ET-1); EN-RAGE; Eotaxin-1; Eotaxin-2; Eotaxin-3; Epidermal Growth Factor (EGF); Epidermal Growth Factor Receptor (EGFR); Epiregulin (EPR); Epithelial cell adhesion molecule (EpCam); Epithelial-Derived Neutrophil-Activating Protein 78 (ENA-78); Erythropoietin (EPO); E-Selectin; Ezrin; Factor VII; Fas Ligand (FasL); FASLG Receptor (FAS); Fatty Acid-Binding Protein, adipocyte (FABP, adipocyte); Fatty Acid-Binding Protein, heart (FABP, heart); Fatty Acid-Binding Protein, liver (FABP, liver); Ferritin (FRTN); Fetuin-A; Fibrinogen; Fibroblast Growth Factor 4 (FGF-4); Fibroblast Growth Factor basic (FGF-basic); Fibulin-1C (Fib-1C); Follicle-Stimulating Hormone (FSH); Galectin-3; Gelsolin; Glucagon; Glucagon-like Peptide 1, total (GLP-1 total); Glucose-6-phosphate Isomerase (G6PI); Glutamate-Cysteine Ligase Regulatory subunit (GCLR); Glutathione S-Transferase alpha (GST-alpha); Glutathione S-Transferase Mu 1 (GST-M1); Granulocyte Colony-Stimulating Factor (G-CSF); Granulocyte-Macrophage Colony-Stimulating Factor (GM-CSF); Growth Hormone (GH); Growth-Regulated alpha protein (GRO-alpha); Haptoglobin; HE4; Heat Shock Protein 60 (HSP-60); Heparin-Binding EGF-Like Growth Factor (HB-EGF); Hepatocyte Growth Factor (HGF); Hepatocyte Growth Factor receptor (HGF receptor)); Hepsin; Human Chorionic Gonadotropin beta (hCG); Human Epidermal Growth Factor Receptor 2 (HER-2); Immunoglobulin A (IgA); Immunoglobulin E (IgE); Immunoglobulin M (IGM); Insulin; Insulin-like Growth Factor Binding Protein 4 (IGFBP4); Insulin-like Growth Factor Binding Protein 5 (IGFBP5); Insulin-like Growth Factor Binding Protein 6 (IGFBP6); Insulin-like Growth Factor I (IGF-I); Insulin-like Growth Factor-Binding Protein 1 (IGFBP-1); Insulin-like Growth Factor-Binding Protein 2 (IGFBP-2); Insulin-like Growth Factor-Binding Protein 3 (IGFBP-3); Intercellular Adhesion Molecule 1 (ICAM-1); Interferon gamma (IFN-gamma); Interferon gamma Induced Protein 10 (IP-10); Interferon-inducible T-cell alpha chemoattractant (ITAC); Interleukin-1 alpha (IL-1 alpha); Interleukin-1 beta (IL-1 beta); Interleukin-1 receptor antagonist (IL-1ra); Interleukin-10 (IL-10); Interleukin-12 Subunit p40 (IL-12p40); Interleukin-12 Subunit p70 (IL-12p70); Interleukin-13 (IL-13); Interleukin-15 (IL-15); Interleukin-16 (IL-16); Interleukin-18 (IL-18); Interleukin-2 (IL-2); Interleukin-2 receptor alpha (IL-2 receptor alpha); Interleukin-25 (IL-25); Interleukin-3 (IL-3); Interleukin-4 (IL-4); Interleukin-5 (IL-5); Interleukin-6 (IL-6); Interleukin-6 receptor (IL-6r); Interleukin-6 receptor subunit beta(IL-6R beta); Interleukin-7 (IL-7); Interleukin-8 (IL-8); Kallikrein 5; Kallikrein-7 (KLK-7); Kidney Injury Molecule-1 (KIM-1); Lactoylglutathione lyase (LGL); Latency-Associated Peptide of Transforming Growth Factor beta 1 (LAP TGF-b1); Lectin-Like Oxidized LDL Receptor 1 (LOX-1); Leptin; Luteinizing Hormone (LH); Lymphotactin; Macrophage Colony-Stimulating Factor 1 (M-CSF); Macrophage inflammatory protein 3 beta (MIP-3 beta); Macrophage Inflammatory Protein-1 alpha (MIP-1 alpha); Macrophage Inflammatory Protein-1 beta (MIP-1 beta); Macrophage Inflammatory Protein-3 alpha (MIP-3 alpha); Macrophage Migration Inhibitory Factor (MIF); Macrophage-Derived Chemokine (MDC); Macrophage-Stimulating Protein (MSP); Malondialdehyde-Modified Low-Density Lipoprotein (MDA-LDL); Maspin; Matrix Metalloproteinase-1 (MMP-1); Matrix Metalloproteinase-10 (MMP-10); Matrix Metalloproteinase-2 (MMP-2); Matrix Metalloproteinase-3 (MMP-3); Matrix Metalloproteinase-7 (MMP-7); Matrix Metalloproteinase-9 (MMP-9); Matrix Metalloproteinase-9, total (MMP-9, total); Mesothelin (MSLN); MHC class I chain-related protein 1 (MICA); Monocyte Chemotactic Protein 1 (MCP-1); Monocyte Chemotactic Protein 2 (MCP-2); Monocyte Chemotactic Protein 3 (MCP-3); Monocyte Chemotactic Protein 4 (MCP-4); Monokine Induced by Gamma Interferon (MIG); Myeloid Progenitor Inhibitory Factor 1 (MPIF-1); Myeloperoxidase (MPO); Myoglobin; Nerve Growth Factor beta (NGF-beta); Neuron Specific Enolase (NSE); Neuronal Cell Adhesion Molecule (Nr-CAM); Neuropilin-1; Neutrophil Gelatinase-Associated Lipocalin (NGAL); N-terminal prohormone of brain natriuretic peptide (NT proBNP); Nucleoside diphosphate kinase B (NDK B); Osteopontin; Osteoprotegerin (OPG); Pancreatic Polypeptide (PPP); Pepsinogen I (PGI); Peroxiredoxin 4 (Prx-IV); Phosphoserine Aminotransferase (PSAT); Placenta Growth Factor (PLGF); Plasminogen Activator Inhibitor 1 (PAI-1); Platelet-Derived Growth Factor BB (PDGF-BB); Pregnancy-Associated Plasma Protein A (PAPP-A); Progesterone; Proinsulin, Intact; Proinsulin, Total; Prolactin (PRL); Prostasin; Prostate-Specific Antigen, Free (PSA-f); Prostatic Acid Phosphatase (PAP); Protein S100-A4 (S100-A4); Protein S100-A6 (S100-A6); Pulmonary and Activation-Regulated Chemokine (PARC); Receptor for advanced glycosylation end products (RAGE); Receptor tyrosine-protein kinase erbB-3 (ErbB3); Resistin; S100 calcium-binding protein B (S100-B); Serotransferrin (Transferrin); Serum Amyloid P-Component (SAP); Serum Glutamic Oxaloacetic Transaminase (SGOT); Sex Hormone-Binding Globulin (SHBG); Sortilin; Squamous Cell Carcinoma Antigen-1 (SCCA-1); Stem Cell Factor (SCF); Stromal cell-derived factor-1 (SDF-1); Superoxide Dismutase 1, Soluble (SOD-1); T Lymphocyte-Secreted Protein I-309 (I-309); Tamm-Horsfall Urinary Glycoprotein (THP); T-Cell-Specific Protein RANTES (RANTES); Tenascin-C (TN-C); Testosterone, Total; Tetranectin; Thrombomodulin (TM); Thrombopoietin; Thrombospondin-1; Thymus-Expressed Chemokine (TECK); Thyroglobulin (TG); Thyroid-Stimulating Hormone (TSH); Thyroxine-Binding Globulin (TBG); Tissue Factor (TF); Tissue Inhibitor of Metalloproteinases 1 (TIMP-1); Tissue type Plasminogen activator (tPA); TNF-Related Apoptosis-Inducing Ligand Receptor 3 (TRAIL-R3); Transforming Growth Factor alpha (TGF-alpha); Transforming Growth Factor beta-3 (TGF-beta-3); Transthyretin (TTR); Trefoil Factor 3 (TFF3); Tumor Necrosis Factor alpha (TNF-alpha); Tumor Necrosis Factor beta (TNF-beta); Tumor Necrosis Factor Receptor 2 (TNFR2); Tumor Necrosis Factor Receptor I (TNF RI); Tyrosine kinase with Ig and EGF homology domains 2 (TIE-2); Urokinase-type Plasminogen Activator (uPA); Urokinase-type Plasminogen Activator Receptor(uPAR); Vascular Cell Adhesion Molecule-1 (VCAM-1); Vascular Endothelial Growth Factor (VEGF); Vascular Endothelial Growth Factor B(VEGF-B); Vascular Endothelial Growth Factor C (VEGF-C); Vascular Endothelial Growth Factor D(VEGF-D); Vascular Endothelial Growth Factor Receptor 1 (VEGFR-1); Vascular Endothelial Growth Factor Receptor 2 (VEGFR-2); Vascular Endothelial Growth Factor Receptor 3 (VEGFR-3); Vitamin K-Dependent Protein S (VKDPS); Vitronectin; von Willebrand Factor (vWF); YKL-40. |
| --- |
