# Supplementary material for: Comprehensive Serum Profiling for the Discovery of Epithelial Ovarian Cancer Biomarkers
Source: PLoS One. 2011 Dec 21;6(12):e29533. doi: 10.1371/journal.pone.0029533 (PMC3244467; doi:10.1371/journal.pone.0029533)
Supplement: Table S2 — Informative Biomarkers with Area Underneath the Curve (AUC) Values Statistically Greater than 0.5. (DOC) [file pone.0029533.s002.doc]

**Supplementary Table 2: Informative Biomarkers with Area Underneath the Curve (AUC) Values Statistically Greater than 0.5.**

| **Up-regulated Markers** | **AUC Value** |
| --- | --- |
| HE4 | 0.933 |
| Cancer Antigen 125 (CA-125) | 0.907 |
| Interleukin-2 receptor alpha (IL-2 receptor alpha) | 0.829 |
| Alpha-1-Antitrypsin (AAT) | 0.817 |
| C-Reactive Protein (CRP) | 0.806 |
| YKL-40 | 0.804 |
| Cellular Fibronectin (cFib) | 0.803 |
| Cancer Antigen 72-4(CA-72-4) | 0.802 |
| Prostasin | 0.800 |
| Tissue Inhibitor of Metalloproteinases 1 (TIMP-1) | 0.797 |
| Interleukin-8 (IL-8) | 0.795 |
| Matrix Metalloproteinase-7 (MMP-7) | 0.787 |
| Interleukin-6 (IL-6) | 0.786 |
| Vascular Endothelial Growth Factor B (VEGF-B) | 0.767 |
| Calprotectin | 0.767 |
| Insulin-like Growth Factor-Binding Protein 2 (IGFBP-2) | 0.759 |
| Lectin-Like Oxidized LDL Receptor 1 (LOX-1) | 0.750 |
| Neuropilin-1 | 0.750 |
| Tumor Necrosis Factor Receptor 2 (TNFR2) | 0.748 |
| Myeloid Progenitor Inhibitory Factor 1 (MPIF-1) | 0.745 |
| Maspin | 0.744 |
| Cancer Antigen 15-3 (CA-15-3) | 0.744 |
| Vascular Endothelial Growth Factor D(VEGF-D) | 0.744 |
| EN-RAGE | 0.742 |
| B cell-activating factor (BAFF) | 0.739 |
| Serum Amyloid P-Component (SAP) | 0.733 |
| Endostatin | 0.731 |
| CD 40 antigen (CD40) | 0.731 |
| Haptoglobin | 0.730 |
| Mesothelin (MSLN) | 0.728 |
| Osteoprotegerin (OPG) | 0.727 |
| Urokinase-type Plasminogen Activator Receptor(uPAR) | 0.726 |
| Growth-Regulated alpha protein (GRO-alpha) | 0.725 |
| Hepatocyte Growth Factor (HGF) | 0.723 |
| Vascular Endothelial Growth Factor (VEGF) | 0.720 |
| Macrophage Inflammatory Protein-1 alpha (MIP-1 alpha) | 0.720 |
| Ferritin (FRTN) | 0.719 |
| von Willebrand Factor (vWF) | 0.718 |
| Pulmonary and Activation-Regulated Chemokine (PARC) | 0.714 |
| Peroxiredoxin 4 (Prx-IV) | 0.706 |
| Tumor Necrosis Factor Receptor I (TNF RI) | 0.705 |
| Insulin-like Growth Factor-Binding Protein 1 (IGFBP-1) | 0.701 |
| Platelet-Derived Growth Factor BB (PDGF-BB) | 0.701 |
| Heparin-Binding EGF-Like Growth Factor (HB-EGF) | 0.699 |
| Cathepsin D | 0.699 |
| MHC class I chain-related protein 1 (MICA) | 0.698 |
| Neutrophil Gelatinase-Associated Lipocalin (NGAL) | 0.697 |
| Sortilin | 0.697 |
| Beta-2-Microglobulin (B2M) | 0.695 |
| Insulin-like Growth Factor Binding Protein 4 (IGFBP4) | 0.694 |
| FASLG Receptor (FAS) | 0.694 |
| Osteopontin | 0.692 |
| Thymus-Expressed Chemokine (TECK) | 0.685 |
| Monokine Induced by Gamma Interferon (MIG) | 0.683 |
| Neuron Specific Enolase (NSE) | 0.683 |
| Plasminogen Activator Inhibitor 1 (PAI-1) | 0.683 |
| Human Chorionic Gonadotropin beta (hCG) | 0.677 |
| Phosphoserine Aminotransferase (PSAT) | 0.677 |
| Interleukin-1 receptor antagonist (IL-1ra) | 0.675 |
| Intercellular Adhesion Molecule 1 (ICAM-1) | 0.675 |
| Myeloperoxidase (MPO) | 0.674 |
| B Lymphocyte Chemoattractant (BLC) | 0.674 |
| Tumor Necrosis Factor alpha (TNF-alpha) | 0.673 |
| Interferon-inducible T-cell alpha chemoattractant (ITAC) | 0.669 |
| Matrix Metalloproteinase-1 (MMP-1) | 0.668 |
| Interleukin-10 (IL-10) | 0.665 |
| Receptor tyrosine-protein kinase erbB-3 (ErbB3) | 0.663 |
| Alpha-1-Antichymotrypsin (AACT) | 0.660 |
| Complement C3 (C3) | 0.657 |
| Thyroxine-Binding Globulin (TBG) | 0.654 |
| Tenascin-C (TN-C) | 0.653 |
| Galectin-3 | 0.652 |
| Trefoil Factor 3 (TFF3) | 0.651 |
| Aldose Reductase | 0.648 |
| Interferon gamma Induced Protein 10 (IP-10) | 0.644 |
| Latency-Associated Peptide of Transforming Growth Factor beta 1 (LAP TGF-b1) | 0.640 |
| Monocyte Chemotactic Protein 3 (MCP-3) | 0.638 |
| Fibrinogen | 0.636 |
| Kallikrein-7 (KLK-7) | 0.634 |
| Hepsin | 0.632 |
| Matrix Metalloproteinase-2 (MMP-2) | 0.629 |
| Vascular Endothelial Growth Factor C (VEGF-C) | 0.629 |
| Interleukin-16 (IL-16) | 0.629 |
| Tissue type Plasminogen activator (tPA) | 0.627 |
| Prostatic Acid Phosphatase (PAP) | 0.627 |
| Angiogenin | 0.624 |
| Sex Hormone-Binding Globulin (SHBG) | 0.623 |
| Epiregulin (EPR) | 0.623 |
| Alpha-1-Microglobulin (A1Micro) | 0.621 |
| Amphiregulin (AR) | 0.620 |
| Collagen IV | 0.619 |
| Matrix Metalloproteinase-10 (MMP-10) | 0.619 |
| Cystatin-C | 0.617 |
| Transforming Growth Factor alpha (TGF-alpha) | 0.617 |
| Vitamin K-Dependent Protein S (VKDPS) | 0.616 |
| N-terminal prohormone of brain natriuretic peptide (NT proBNP) | 0.615 |
| Macrophage inflammatory protein 3 beta (MIP-3 beta) | 0.615 |
| Kidney Injury Molecule-1 (KIM-1) | 0.613 |
| Fatty Acid-Binding Protein, adipocyte (FABP, adipocyte) | 0.613 |
| Angiopoietin-2 (ANG-2) | 0.612 |
| Calcitonin | 0.611 |
| Complement Factor H | 0.611 |
| Human Epidermal Growth Factor Receptor 2 (HER-2) | 0.605 |
| Fatty Acid-Binding Protein, heart (FABP, heart) | 0.605 |
| Monocyte Chemotactic Protein 1 (MCP-1) | 0.605 |
| Apolipoprotein E (Apo E) | 0.605 |
| Macrophage Migration Inhibitory Factor (MIF) | 0.601 |
| TNF-Related Apoptosis-Inducing Ligand Receptor 3 (TRAIL-R3) | 0.601 |
| Placenta Growth Factor (PLGF) | 0.600 |
| Luteinizing Hormone (LH) | 0.599 |
| Resistin | 0.599 |
| Matrix Metalloproteinase-9, total (MMP-9, total) | 0.599 |
| Follicle-Stimulating Hormone (FSH) | 0.593 |
| Fibroblast Growth Factor basic (FGF-basic) | 0.591 |
| CD40 Ligand (CD40-L) | 0.591 |
| Macrophage Inflammatory Protein-1 beta (MIP-1 beta) | 0.590 |
| Chemokine CC-4 (HCC-4) | 0.585 |
| Apolipoprotein(a) (Lp(a)) | 0.585 |
| Growth Hormone (GH) | 0.583 |
| Vascular Endothelial Growth Factor Receptor 3 (VEGFR-3) | 0.582 |
| 6Ckine | 0.582 |
| Carcinoembryonic Antigen (CEA) | 0.581 |
| Alpha-Fetoprotein (AFP) | 0.580 |
| Erythropoietin (EPO) | 0.579 |
| Cortisol (Cortisol) | 0.578 |
| Bone Morphogenetic Protein 6 (BMP-6) | 0.578 |
| Epidermal Growth Factor (EGF) | 0.578 |
| Monocyte Chemotactic Protein 2 (MCP-2) | 0.576 |
| Vascular Cell Adhesion Molecule-1 (VCAM-1) | 0.576 |
| T-Cell-Specific Protein RANTES (RANTES) | 0.575 |
| S100 calcium-binding protein B (S100-B) | 0.570 |
| Chromogranin-A (CgA) | 0.569 |
| Matrix Metalloproteinase-3 (MMP-3) | 0.567 |
| T Lymphocyte-Secreted Protein I-309 (I-309) | 0.563 |
| Pancreatic Polypeptide (PPP) | 0.560 |
| Pepsinogen I (PGI) | 0.559 |

| **Down-regulated Biomarkers** | **AUC Value** |
| --- | --- |
| Transthyretin (TTR) | 0.745 |
| Apolipoprotein A-IV (Apo A-IV) | 0.713 |
| Leptin | 0.664 |
| Serotransferrin (Transferrin) | 0.660 |
| Fetuin-A | 0.644 |
| Epidermal Growth Factor Receptor (EGFR) | 0.635 |
| Apolipoprotein A-II (Apo A-II) | 0.631 |
| Endoglin | 0.627 |
| Insulin | 0.626 |
| Gelsolin | 0.613 |
| Angiotensin-Converting Enzyme (ACE) | 0.611 |
| Vascular Endothelial Growth Factor Receptor 2 (VEGFR-2) | 0.611 |
| Tetranectin | 0.608 |
| Interleukin-13 (IL-13) | 0.605 |
| Interleukin-1 alpha (IL-1 alpha) | 0.602 |
| Thrombopoietin | 0.599 |
| E-Selectin | 0.597 |
| Tamm-Horsfall Urinary Glycoprotein (THP) | 0.595 |
| Glucagon-like Peptide 1, total (GLP-1 total) | 0.591 |
| Alpha-2-Macroglobulin (A2Macro) | 0.591 |
| Granulocyte Colony-Stimulating Factor (G-CSF) | 0.589 |
| Brain-Derived Neurotrophic Factor (BDNF) | 0.586 |
| Interleukin-3 (IL-3) | 0.583 |
| Fibulin-1C (Fib-1C) | 0.580 |
| Macrophage-Derived Chemokine (MDC) | 0.580 |
| Interleukin-2 (IL-2) | 0.578 |
| Superoxide Dismutase 1, Soluble (SOD-1) | 0.578 |
| Receptor for advanced glycosylation end products (RAGE) | 0.575 |
| Interleukin-7 (IL-7) | 0.574 |
| Glutathione S-Transferase alpha (GST-alpha) | 0.573 |
| Thyroglobulin (TG) | 0.573 |
| Interleukin-15 (IL-15) | 0.573 |
| Vascular Endothelial Growth Factor Receptor 1 (VEGFR-1) | 0.573 |
| Tumor Necrosis Factor beta (TNF-beta) | 0.573 |
| Interleukin-5 (IL-5) | 0.564 |
| Progesterone | 0.563 |
| Monocyte Chemotactic Protein 4 (MCP-4) | 0.562 |
| Immunoglobulin E (IgE) | 0.558 |
| Insulin-like Growth Factor Binding Protein 6 (IGFBP6) | 0.556 |
