# Supplementary material for: Comprehensive Serum Profiling for the Discovery of Epithelial Ovarian Cancer Biomarkers
Source: PLoS One. 2011 Dec 21;6(12):e29533. doi: 10.1371/journal.pone.0029533 (PMC3244467; doi:10.1371/journal.pone.0029533)
Supplement: Table S3 — Identity of Markers in Clusters A Through D. (DOC) [file pone.0029533.s003.doc]

**Supplementary Table 3: Identity of Markers in Clusters A Through D.**

| **Cluster** | **ID** | **Markers** |
| --- | --- | --- |
| **A** | 1-10 | Matrix metalloproteinase-10, TNF-Related Apoptosis-Inducing Ligand Receptor 3, FASLG Receptor (FAS), CA 72-4, monokine induced by gamma interferon, hepatocyte growth factor, CA 15-3, human epidermal growth factor receptor 2 (HER-2), thymus-expressed chemokine, monocyte chemotactic protein 3, and myeloid progenitor inhibitory factor 1. |
| **B** | 58-67 | Adipocyte fatty acid binding protein (FABP), galectin-3, endostatin, heart FABP, tumor necrosis factor receptor 2 (TNFR2), TNFR1, CD40 antigen, insulin-like growth factor binding protein 4, β2-Microglobulin, and cystatin C. |
|  | 68-69 | Weak association  α-1-Microglobulin and hepsin |
| **C** | 81-87 | Kallikrein 7, CA-125, prostasin, vascular endothelial growth factor-B (VEGF-B), VEGF-D, maspin and mesothelin |
|  | 79-80 | Weak association  HE4 and urokinase-type plasminogen activator receptor |
| **D** | 32-55 | Calprotectin, EN-RAGE, IL-16, neutrophil gelatinase-associated lipocalin, lectin-like oxidized LDL receptor 1, myeloperoxidase, ferritin, IL-1 receptor α, peroxiredoxin 4, IL-6, tenascin C, osteoprotegrin, YKL-40, von Willebrand Factor, cellular fibronectin, pulmonary and activation-regulated chemokine, neuropilin-1, haptoglobin, α1-antitrypsin, plasminogen activator inhibitor 1, tissue inhibitor of metalloproteinases 1 (TIMP-1), VEGF, C-reactive protein and IL-2 receptor α. |
