# Supplementary material for: Comprehensive Serum Profiling for the Discovery of Epithelial Ovarian Cancer Biomarkers
Source: PLoS One. 2011 Dec 21;6(12):e29533. doi: 10.1371/journal.pone.0029533 (PMC3244467; doi:10.1371/journal.pone.0029533)
Supplement: Table S4 — Correlation of Markers in Cluster A. (DOC) [file pone.0029533.s004.doc]

**Supplementary Table 4: Correlation of Markers in Cluster A.**

|  | MMP-10 | TRAILR-3 | FAS | CA 72-4 | MIG | HGF | CA 15-3 | HER-2 | TEC | MCP 3 | MPIF-1 |
| --- | --- | --- | --- | --- | --- | --- | --- | --- | --- | --- | --- |
| MMP-10 | 1.000 | 0.232 | 0.179 | 0.215 | 0.223 | 0.269 | 0.323 | 0.258 | 0.251 | 0.189 | 0.234 |
| TRAILR-3 | 0.232 | 1.000 | 0.547 | 0.373 | 0.528 | 0.620 | 0.580 | 0.575 | 0.621 | 0.545 | 0.651 |
| FAS | 0.179 | 0.547 | 1.000 | 0.487 | 0.600 | 0.686 | 0.602 | 0.662 | 0.653 | 0.619 | 0.654 |
| CA 72-4 | 0.215 | 0.373 | 0.487 | 1.000 | 0.533 | 0.606 | 0.793 | 0.701 | 0.621 | 0.628 | 0.616 |
| MIG | 0.223 | 0.528 | 0.600 | 0.533 | 1.000 | 0.728 | 0.783 | 0.799 | 0.810 | 0.797 | 0.830 |
| HGF | 0.269 | 0.620 | 0.686 | 0.606 | 0.728 | 1.000 | 0.797 | 0.843 | 0.881 | 0.760 | 0.829 |
| CA 15-3 | 0.323 | 0.580 | 0.602 | 0.793 | 0.783 | 0.797 | 1.000 | 0.921 | 0.913 | 0.827 | 0.851 |
| HER-2 | 0.258 | 0.575 | 0.662 | 0.701 | 0.799 | 0.843 | 0.921 | 1.000 | 0.948 | 0.873 | 0.878 |
| TEC | 0.251 | 0.621 | 0.653 | 0.621 | 0.810 | 0.881 | 0.913 | 0.948 | 1.000 | 0.853 | 0.888 |
| MCP 3 | 0.189 | 0.545 | 0.619 | 0.628 | 0.797 | 0.760 | 0.827 | 0.873 | 0.853 | 1.000 | 0.914 |
| MPIF-1 | 0.234 | 0.651 | 0.654 | 0.616 | 0.830 | 0.829 | 0.851 | 0.878 | 0.888 | 0.914 | 1.000 |

Abbreviations: MMP-10, Matrix metalloproteinase-10; TRAILR-3, TNF-Related Apoptosis-Inducing Ligand Receptor 3; FAS, FASLG Receptor; MIG, monokine induced by gamma interferon; HGF, hepatocyte growth factor; HER-2, human epidermal growth factor receptor 2; TEC, thymus-expressed chemokine; MCP-3, monocyte chemotactic protein 3; MPIF-1, myeloid progenitor inhibitory factor.
