# Supplementary material for: Comprehensive Serum Profiling for the Discovery of Epithelial Ovarian Cancer Biomarkers
Source: PLoS One. 2011 Dec 21;6(12):e29533. doi: 10.1371/journal.pone.0029533 (PMC3244467; doi:10.1371/journal.pone.0029533)
Supplement: Table S5 — Correlation of Markers in Cluster B. (DOC) [file pone.0029533.s005.doc]

**Supplementary Table 5: Correlation of Markers in Cluster B.**

|  | aFABP | Galectin-3 | Endostatin | hFABP | TNFR2 | TNFR1 | CD40a | IGFBP4 | β2M | Cystatin C | α1M | Hepsin |
| --- | --- | --- | --- | --- | --- | --- | --- | --- | --- | --- | --- | --- |
| aFABP | 1.000 | 0.626 | 0.613 | 0.619 | 0.667 | 0.623 | 0.668 | 0.659 | 0.697 | 0.533 | 0.451 | 1.000 |
| Galectin-3 | 0.626 | 1.000 | 0.655 | 0.736 | 0.642 | 0.680 | 0.718 | 0.703 | 0.706 | 0.509 | 0.556 | 0.626 |
| Endostatin | 0.613 | 0.655 | 1.000 | 0.743 | 0.705 | 0.729 | 0.810 | 0.745 | 0.773 | 0.574 | 0.672 | 0.613 |
| hFABP | 0.572 | 0.589 | 0.586 | 0.733 | 0.787 | 0.837 | 0.755 | 0.838 | 0.811 | 0.482 | 0.429 | 0.572 |
| TNFR2 | 0.619 | 0.736 | 0.743 | 1.000 | 0.817 | 0.826 | 0.821 | 0.884 | 0.808 | 0.591 | 0.592 | 0.619 |
| TNFR1 | 0.667 | 0.642 | 0.705 | 0.817 | 1.000 | 0.856 | 0.835 | 0.851 | 0.830 | 0.535 | 0.505 | 0.667 |
| CD40a | 0.623 | 0.680 | 0.729 | 0.826 | 0.856 | 1.000 | 0.843 | 0.887 | 0.870 | 0.552 | 0.539 | 0.623 |
| IGFBP4 | 0.668 | 0.718 | 0.810 | 0.821 | 0.835 | 0.843 | 1.000 | 0.868 | 0.905 | 0.624 | 0.646 | 0.668 |
| β2M | 0.659 | 0.703 | 0.745 | 0.884 | 0.851 | 0.887 | 0.868 | 1.000 | 0.917 | 0.587 | 0.555 | 0.659 |
| Cystatin C | 0.697 | 0.706 | 0.773 | 0.808 | 0.830 | 0.870 | 0.905 | 0.917 | 1.000 | 0.617 | 0.586 | 0.697 |
| α1M | 0.533 | 0.509 | 0.574 | 0.591 | 0.535 | 0.552 | 0.624 | 0.587 | 0.617 | 1.000 | 0.634 | 0.533 |
| Hepsin | 0.451 | 0.556 | 0.672 | 0.592 | 0.505 | 0.539 | 0.646 | 0.555 | 0.586 | 0.634 | 1.000 | 0.451 |

Abbreviations: aFABP, Adipocyte fatty acid binding protein; hFABP, Heart FABP; TNFR2, Tumor necrosis factor receptor 2; TNFR1, Tumor necrosis factor receptor 1; CD40a, CD40 antigen; IGFBP4, Insulin-like growth factor binding protein 4; β2M, β2-Microglobulin; α1M, α-1-Microglobulin.
