# Supplementary material for: Comprehensive Serum Profiling for the Discovery of Epithelial Ovarian Cancer Biomarkers
Source: PLoS One. 2011 Dec 21;6(12):e29533. doi: 10.1371/journal.pone.0029533 (PMC3244467; doi:10.1371/journal.pone.0029533)
Supplement: Table S6 — Correlation of Markers in Cluster C. (DOC) [file pone.0029533.s006.doc]

**Supplementary Table 6: Correlation of Markers in Cluster C.**

|  | KLK7 | CA-125 | Prostasin | VEGF-B | VEBF-D | Maspin | Mesothelin | HE4 | uPAR |
| --- | --- | --- | --- | --- | --- | --- | --- | --- | --- |
| KLK7 | 1.000 | 0.507 | 0.482 | 0.539 | 0.530 | 0.594 | 0.590 | 0.338 | 0.222 |
| CA-125 | 0.507 | 1.000 | 0.551 | 0.505 | 0.568 | 0.599 | 0.600 | 0.280 | 0.358 |
| Prostasin | 0.482 | 0.551 | 1.000 | 0.709 | 0.757 | 0.748 | 0.639 | 0.463 | 0.549 |
| VEBF-B | 0.539 | 0.505 | 0.709 | 1.000 | 0.745 | 0.770 | 0.718 | 0.413 | 0.391 |
| VEBF-D | 0.530 | 0.568 | 0.757 | 0.745 | 1.000 | 0.821 | 0.808 | 0.409 | 0.453 |
| Maspin | 0.594 | 0.599 | 0.748 | 0.770 | 0.821 | 1.000 | 0.878 | 0.517 | 0.434 |
| Mesothelin | 0.590 | 0.600 | 0.639 | 0.718 | 0.808 | 0.878 | 1.000 | 0.335 | 0.377 |
| HE4 | 0.338 | 0.280 | 0.463 | 0.413 | 0.409 | 0.517 | 0.335 | 1.000 | 0.303 |
| uPAR | 0.222 | 0.358 | 0.549 | 0.391 | 0.453 | 0.434 | 0.377 | 0.303 | 1.000 |

Abbreviations: KLK7, Kallikrein 7; VEGF-B, vascular endothelial growth factor-B; VEGF-D, vascular endothelial growth factor-D.
