# Supplementary material for: Comprehensive Serum Profiling for the Discovery of Epithelial Ovarian Cancer Biomarkers
Source: PLoS One. 2011 Dec 21;6(12):e29533. doi: 10.1371/journal.pone.0029533 (PMC3244467; doi:10.1371/journal.pone.0029533)
Supplement: Table S7 — Correlation of Markers in Cluster D. (DOC) [file pone.0029533.s007.doc]

**Supplementary Table 7: Correlation of Markers in Cluster D.**

|  | Calpro | ENRAGE | IL-16 | N-GAL | LOX-1 | Myelo | Ferritin | IL1-Rα | PER-4 | IL-6 | Ten C | Osteop | YKL-40 | vWF | cFib | PARC | Neu-1 | Hapto | AAT | PAI-1 | TIMP-1 | VEGF | CRP | IL2-Rα |
| --- | --- | --- | --- | --- | --- | --- | --- | --- | --- | --- | --- | --- | --- | --- | --- | --- | --- | --- | --- | --- | --- | --- | --- | --- |
| Calpro | 1.000 | 0.711 | 0.305 | 0.472 | 0.402 | 0.406 | 0.523 | 0.486 | 0.494 | 0.396 | 0.234 | 0.153 | 0.394 | 0.312 | 0.189 | 0.234 | 0.341 | 0.299 | 0.305 | 0.203 | 0.311 | 0.341 | 0.275 | 1.000 |
| ENRAGE | 0.711 | 1.000 | 0.500 | 0.622 | 0.572 | 0.612 | 0.449 | 0.368 | 0.475 | 0.406 | 0.292 | 0.204 | 0.390 | 0.322 | 0.317 | 0.310 | 0.447 | 0.422 | 0.454 | 0.323 | 0.465 | 0.501 | 0.365 | 0.711 |
| IL-16 | 0.305 | 0.500 | 1.000 | 0.606 | 0.545 | 0.505 | 0.337 | 0.401 | 0.330 | 0.195 | 0.227 | 0.298 | 0.447 | 0.382 | 0.337 | 0.404 | 0.309 | 0.208 | 0.266 | 0.178 | 0.356 | 0.337 | 0.421 | 0.305 |
| N-GAL | 0.472 | 0.622 | 0.606 | 1.000 | 0.734 | 0.621 | 0.392 | 0.278 | 0.364 | 0.307 | 0.329 | 0.291 | 0.438 | 0.361 | 0.323 | 0.432 | 0.419 | 0.317 | 0.424 | 0.314 | 0.489 | 0.477 | 0.521 | 0.472 |
| LOX-1 | 0.402 | 0.572 | 0.545 | 0.734 | 1.000 | 0.775 | 0.344 | 0.332 | 0.428 | 0.322 | 0.302 | 0.309 | 0.424 | 0.328 | 0.315 | 0.406 | 0.417 | 0.381 | 0.439 | 0.336 | 0.473 | 0.486 | 0.490 | 0.402 |
| Myelo | 0.406 | 0.612 | 0.505 | 0.621 | 0.775 | 1.000 | 0.325 | 0.301 | 0.353 | 0.281 | 0.234 | 0.223 | 0.316 | 0.311 | 0.221 | 0.322 | 0.341 | 0.360 | 0.392 | 0.334 | 0.425 | 0.455 | 0.359 | 0.406 |
| Ferritin | 0.523 | 0.449 | 0.337 | 0.392 | 0.344 | 0.325 | 1.000 | 0.392 | 0.530 | 0.367 | 0.242 | 0.415 | 0.426 | 0.334 | 0.320 | 0.386 | 0.373 | 0.306 | 0.370 | 0.283 | 0.456 | 0.452 | 0.427 | 0.523 |
| IL1-Rα | 0.486 | 0.368 | 0.401 | 0.278 | 0.332 | 0.301 | 0.392 | 1.000 | 0.543 | 0.241 | 0.210 | 0.181 | 0.491 | 0.355 | 0.136 | 0.203 | 0.203 | 0.215 | 0.188 | 0.208 | 0.226 | 0.220 | 0.176 | 0.486 |
| PER-4 | 0.494 | 0.475 | 0.330 | 0.364 | 0.428 | 0.353 | 0.530 | 0.543 | 1.000 | 0.468 | 0.430 | 0.416 | 0.513 | 0.463 | 0.370 | 0.425 | 0.448 | 0.423 | 0.469 | 0.361 | 0.525 | 0.492 | 0.454 | 0.494 |
| IL-6 | 0.396 | 0.406 | 0.195 | 0.307 | 0.322 | 0.281 | 0.367 | 0.241 | 0.468 | 1.000 | 0.402 | 0.167 | 0.330 | 0.309 | 0.297 | 0.295 | 0.375 | 0.444 | 0.412 | 0.294 | 0.428 | 0.500 | 0.328 | 0.396 |
| Ten C | 0.234 | 0.292 | 0.227 | 0.329 | 0.302 | 0.234 | 0.242 | 0.210 | 0.430 | 0.402 | 1.000 | 0.212 | 0.351 | 0.375 | 0.342 | 0.324 | 0.365 | 0.357 | 0.423 | 0.271 | 0.396 | 0.382 | 0.390 | 0.234 |
| Osteop | 0.153 | 0.204 | 0.298 | 0.291 | 0.309 | 0.223 | 0.415 | 0.181 | 0.416 | 0.167 | 0.212 | 1.000 | 0.287 | 0.207 | 0.269 | 0.253 | 0.356 | 0.128 | 0.273 | 0.187 | 0.334 | 0.234 | 0.471 | 0.153 |
| YKL-40 | 0.394 | 0.390 | 0.447 | 0.438 | 0.424 | 0.316 | 0.426 | 0.491 | 0.513 | 0.330 | 0.351 | 0.287 | 1.000 | 0.511 | 0.412 | 0.447 | 0.422 | 0.466 | 0.436 | 0.286 | 0.526 | 0.474 | 0.529 | 0.394 |
| vWF | 0.312 | 0.322 | 0.382 | 0.361 | 0.328 | 0.311 | 0.334 | 0.355 | 0.463 | 0.309 | 0.375 | 0.207 | 0.511 | 1.000 | 0.425 | 0.409 | 0.451 | 0.398 | 0.355 | 0.230 | 0.482 | 0.454 | 0.456 | 0.312 |
| cFib | 0.189 | 0.317 | 0.337 | 0.323 | 0.315 | 0.221 | 0.320 | 0.136 | 0.370 | 0.297 | 0.342 | 0.269 | 0.412 | 0.425 | 1.000 | 0.409 | 0.454 | 0.430 | 0.449 | 0.216 | 0.532 | 0.517 | 0.618 | 0.189 |
| PARC | 0.234 | 0.310 | 0.404 | 0.432 | 0.406 | 0.322 | 0.386 | 0.203 | 0.425 | 0.295 | 0.324 | 0.253 | 0.447 | 0.409 | 0.409 | 1.000 | 0.404 | 0.429 | 0.380 | 0.359 | 0.540 | 0.562 | 0.552 | 0.234 |
| Neu-1 | 0.341 | 0.447 | 0.309 | 0.419 | 0.417 | 0.341 | 0.373 | 0.203 | 0.448 | 0.375 | 0.365 | 0.356 | 0.422 | 0.451 | 0.454 | 0.404 | 1.000 | 0.496 | 0.497 | 0.378 | 0.602 | 0.494 | 0.532 | 0.341 |
| Hapto | 0.299 | 0.422 | 0.208 | 0.317 | 0.381 | 0.360 | 0.306 | 0.215 | 0.423 | 0.444 | 0.357 | 0.128 | 0.466 | 0.398 | 0.430 | 0.429 | 0.496 | 1.000 | 0.606 | 0.543 | 0.641 | 0.597 | 0.470 | 0.299 |
| AAT | 0.305 | 0.454 | 0.266 | 0.424 | 0.439 | 0.392 | 0.370 | 0.188 | 0.469 | 0.412 | 0.423 | 0.273 | 0.436 | 0.355 | 0.449 | 0.380 | 0.497 | 0.606 | 1.000 | 0.602 | 0.708 | 0.592 | 0.563 | 0.305 |
| PAI-1 | 0.203 | 0.323 | 0.178 | 0.314 | 0.336 | 0.334 | 0.283 | 0.208 | 0.361 | 0.294 | 0.271 | 0.187 | 0.286 | 0.230 | 0.216 | 0.359 | 0.378 | 0.543 | 0.602 | 1.000 | 0.761 | 0.488 | 0.448 | 0.203 |
| TIMP-1 | 0.311 | 0.465 | 0.356 | 0.489 | 0.473 | 0.425 | 0.456 | 0.226 | 0.525 | 0.428 | 0.396 | 0.334 | 0.526 | 0.482 | 0.532 | 0.540 | 0.602 | 0.641 | 0.708 | 0.761 | 1.000 | 0.662 | 0.693 | 0.311 |
| VEGF | 0.290 | 0.363 | 0.343 | 0.520 | 0.431 | 0.396 | 0.334 | 0.167 | 0.445 | 0.441 | 0.402 | 0.275 | 0.411 | 0.348 | 0.375 | 0.491 | 0.446 | 0.456 | 0.506 | 0.506 | 0.648 | 0.525 | 0.609 | 0.290 |
| CRP | 0.341 | 0.501 | 0.337 | 0.477 | 0.486 | 0.455 | 0.452 | 0.220 | 0.492 | 0.500 | 0.382 | 0.234 | 0.474 | 0.454 | 0.517 | 0.562 | 0.494 | 0.597 | 0.592 | 0.488 | 0.662 | 1.000 | 0.619 | 0.341 |
| IL2-Rα | 0.275 | 0.365 | 0.421 | 0.521 | 0.490 | 0.359 | 0.427 | 0.176 | 0.454 | 0.328 | 0.390 | 0.471 | 0.529 | 0.456 | 0.618 | 0.552 | 0.532 | 0.470 | 0.563 | 0.448 | 0.693 | 0.619 | 1.000 | 0.275 |

Abbreviations: Calpro, Calprotectin; N-GAL, Neutrophil gelatinase-associated lipocalin; LOX-1, Lectin-like oxidized LDL receptor 1; Myelo, Myeloperoxidase; IL1-Rα, IL-1 receptor α;  PER-4, Peroxiredoxin 4; Ten C, Tenascin C; Osteop, Osteoprotegrin; vWF, von Willebrand Factor; cFib, cellular fibronectin; PARC, pulmonary and activation-regulated chemokine; Neu-1, Neuropilin-1; Hapto, Haptoglobin; ATT, α1-antitrypsin; PAI-1, Plasminogen activator inhibitor 1; TIMP-1, Tissue inhibitor of metalloproteinases 1 (TIMP-1); CRP, C-reactive protein; IL2-Rα, IL-2 receptor α.
