# Supplementary material for: Comprehensive Serum Profiling for the Discovery of Epithelial Ovarian Cancer Biomarkers
Source: PLoS One. 2011 Dec 21;6(12):e29533. doi: 10.1371/journal.pone.0029533 (PMC3244467; doi:10.1371/journal.pone.0029533)
Supplement: Table S8 — Area Underneath the Curve (AUC) values from Receiver Operating Characteristic (ROC) curve analysis of the top 20 markers broken out by menopausal status. (DOC) [file pone.0029533.s008.doc]

**Supplementary Table 8.** Area Underneath the Curve (AUC) values from Receiver Operating Characteristic (ROC) curve analysis of the top 20 markers broken out by menopausal status.

| **Marker** | **All** | **95% CI** | **Pre** | **95% CI** | **Post** | **95% CI** |
| --- | --- | --- | --- | --- | --- | --- |
| **HE4** | 0.933 | 0.905-0.961 | 0.912 | 0.826-0.997 | 0.927 | 0.895-0.959 |
| **CA-125** | 0.907 | 0.877-0.938 | 0.907 | 0.849-0.966 | 0.927 | 0.896-0.959 |
| **IL-2 receptor alpha** | 0.829 | 0.790-0.868 | 0.812 | 0.724-0.899 | 0.824 | 0.778-0.870 |
| **Alpha-1-antitrypsin** | 0.817 | 0.773-0.861 | 0.876 | 0.806-0.945 | 0.818 | 0.768-0.869 |
| **C-reactive protein** | 0.806 | 0.763-0.850 | 0.839 | 0.752-0.926 | 0.797 | 0.745-0.849 |
| **YKL-40** | 0.804 | 0.763-0.845 | 0.824 | 0.734-0.913 | 0.761 | 0.708-0.814 |
| **Cellular Fibronectin** | 0.803 | 0.760-0.846 | 0.835 | 0.747-0.924 | 0.776 | 0.723-0.830 |
| **CA-72-4** | 0.802 | 0.753-0.850 | 0.769 | 0.650-0.888 | 0.806 | 0.752-0.861 |
| **Prostasin** | 0.800 | 0.755-0.845 | 0.732 | 0.609-0.855 | 0.799 | 0.748-0.850 |
| **TIMP-1** | 0.797 | 0.751-0.844 | 0.767 | 0.664-0.870 | 0.797 | 0.744-0.851 |
| **IL-8** | 0.795 | 0.752-0.837 | 0.823 | 0.736-0.910 | 0.755 | 0.700-0.810 |
| **MMP-7** | 0.787 | 0.741-0.834 | 0.757 | 0.644-0.870 | 0.771 | 0.717-0.826 |
| **IL-6** | 0.786 | 0.740-0.833 | 0.791 | 0.683-0.898 | 0.778 | 0.724-0.833 |
| **VEGF-B** | 0.767 | 0.720-0.815 | 0.704 | 0.581-0.826 | 0.778 | 0.725-0.832 |
| **Calprotectin** | 0.767 | 0.719-0.814 | 0.795 | 0.698-0.892 | 0.772 | 0.718-0.827 |
| **IGFBP-2** | 0.759 | 0.714-0.805 | 0.799 | 0.692-0.906 | 0.713 | 0.656-0.769 |
| **LOX-1** | 0.750 | 0.704-0.796 | 0.734 | 0.624-0.844 | 0.763 | 0.711-0.816 |
| **Neuropilin-1** | 0.750 | 0.702-0.798 | 0.789 | 0.699-0.879 | 0.727 | 0.669-0.784 |
| **TNFR2** | 0.748 | 0.700-0.796 | 0.684 | 0.561-0.806 | 0.735 | 0.679-0.791 |
| **MPIF-1** | 0.745 | 0.697-0.793 | 0.803 | 0.709-0.896 | 0.716 | 0.658-0.774 |

Abbreviations: Pre, Pre-menopausal; Post, Post-menopausal; HE4, human epididymis protein-4; CA, cancer antigen; TIMP-1, tissue inhibitor of metalloproteinases 1; IL, interleukin; MMP-7, Matrix Metalloproteinase-7; VEGF-B, vascular endothelial growth factor B; IGFBP-2, insulin-like growth factor-binding protein 2; LOX-1, lectin-like oxidized LDL receptor 1; TNFR2, tumor necrosis factor receptor 2; MPIF-1, myeloid progenitor inhibitory factor 1.
